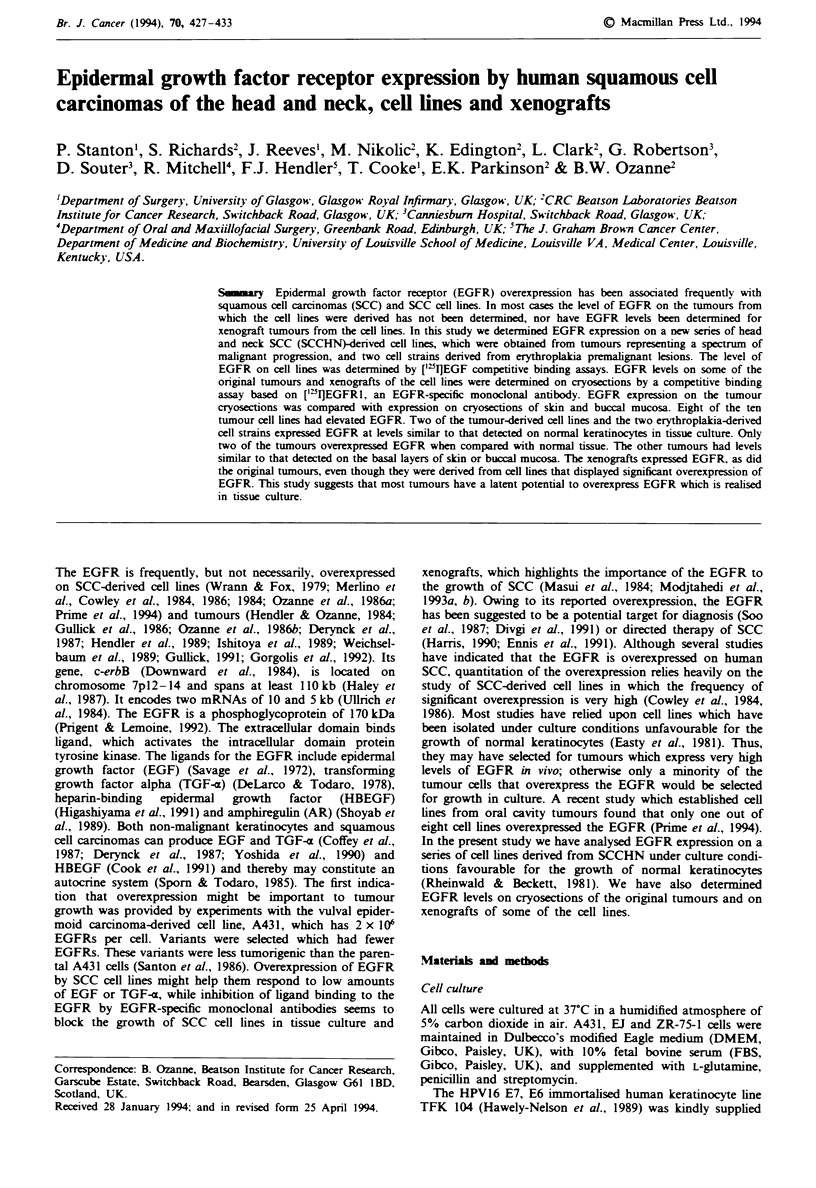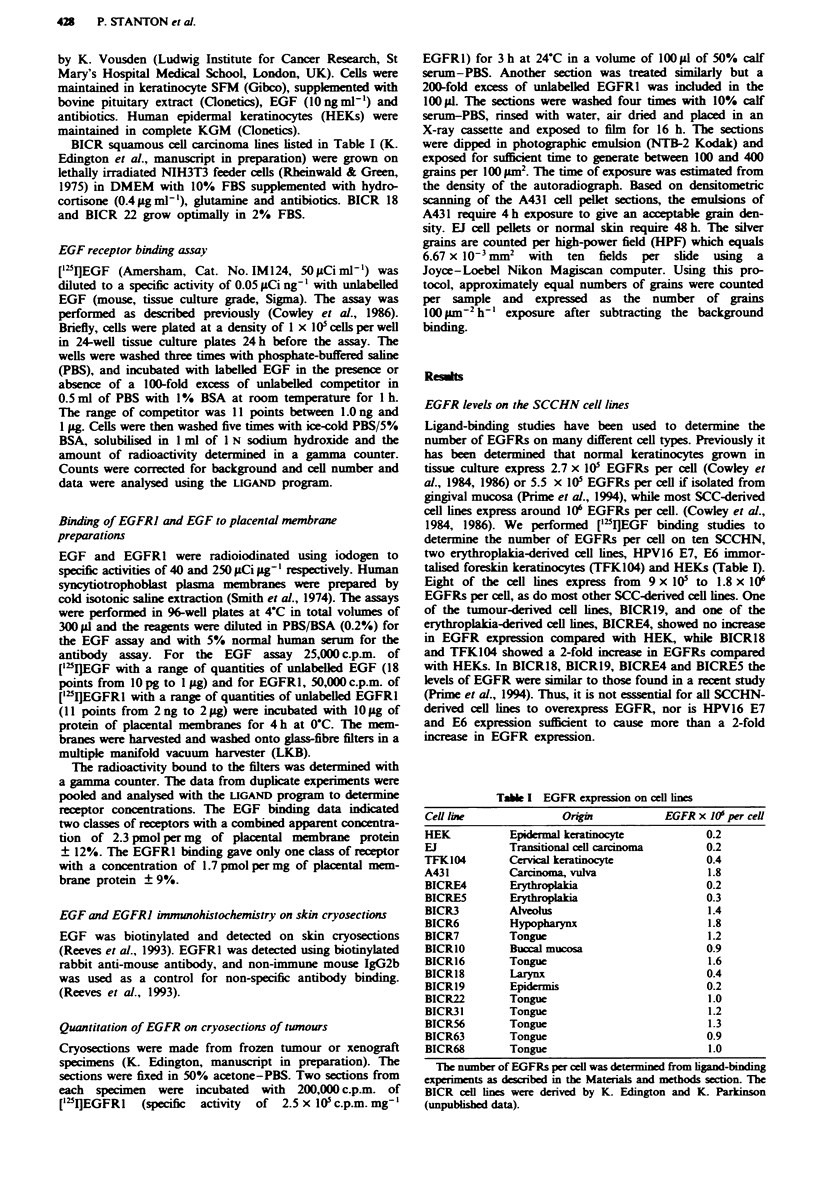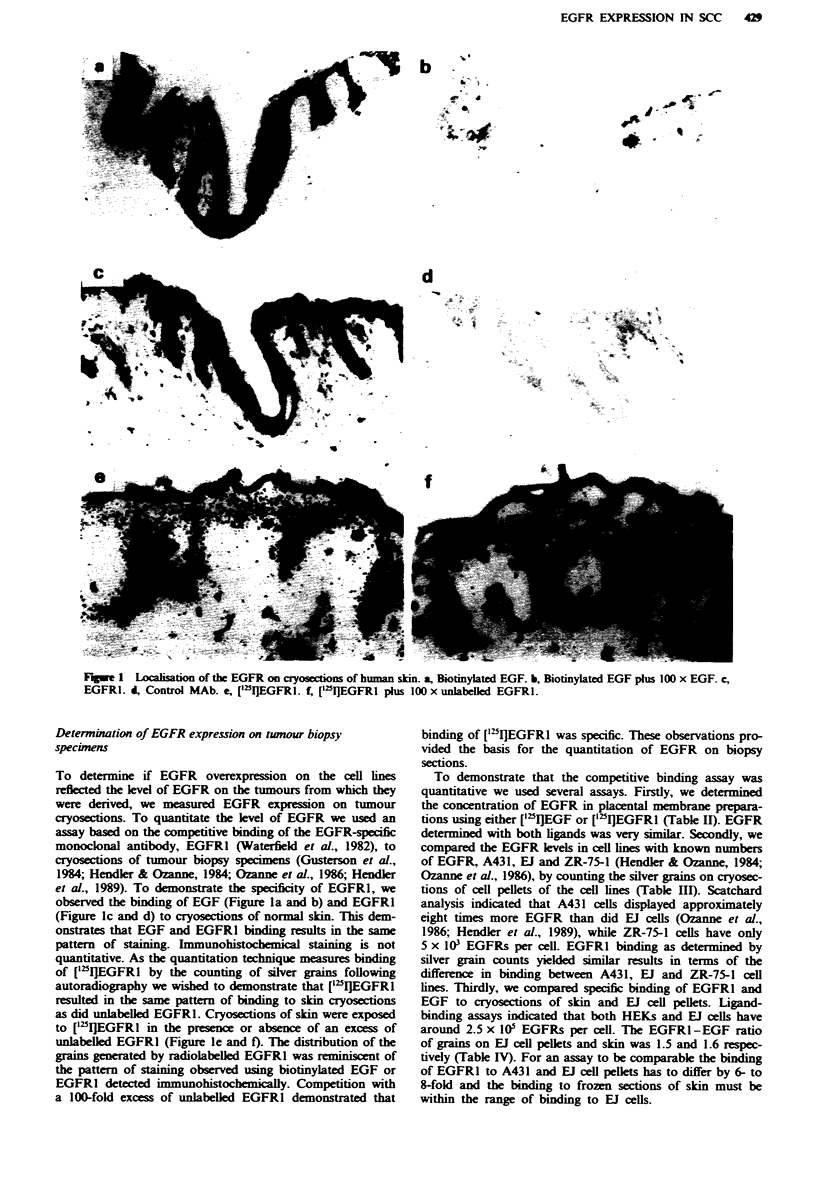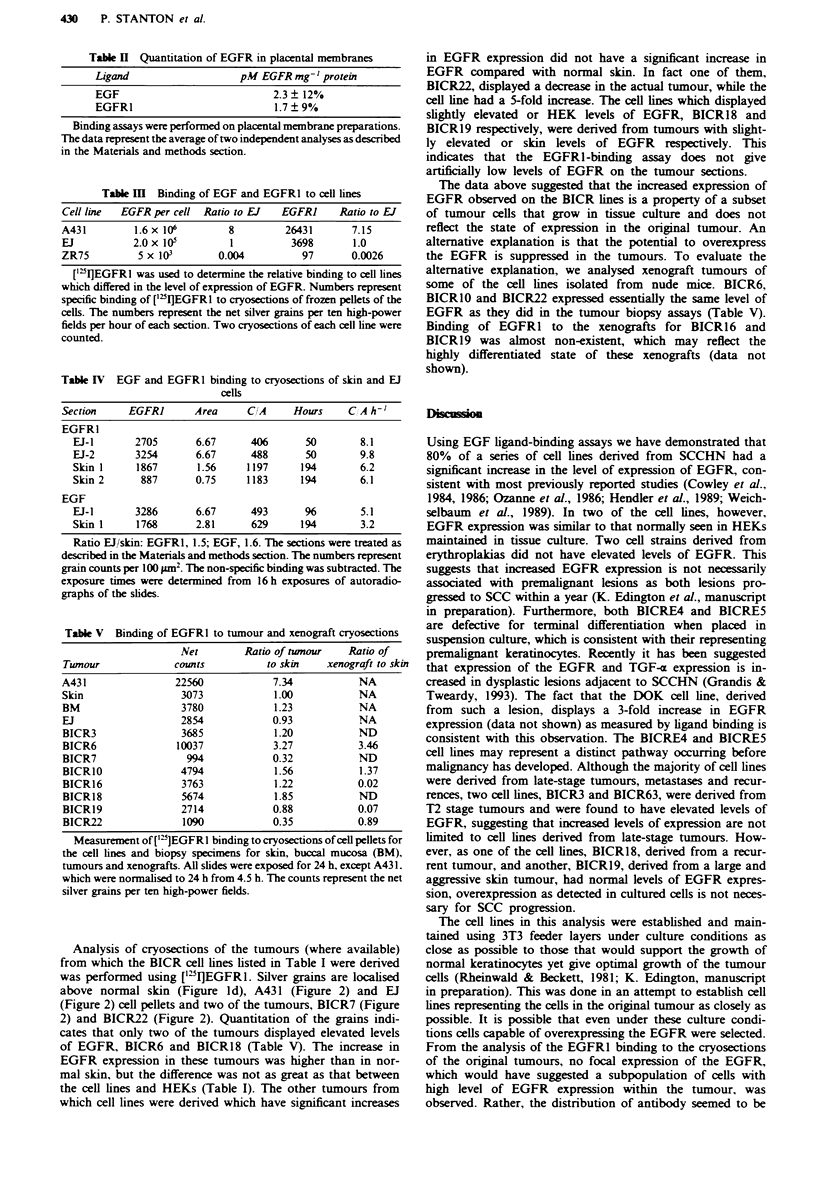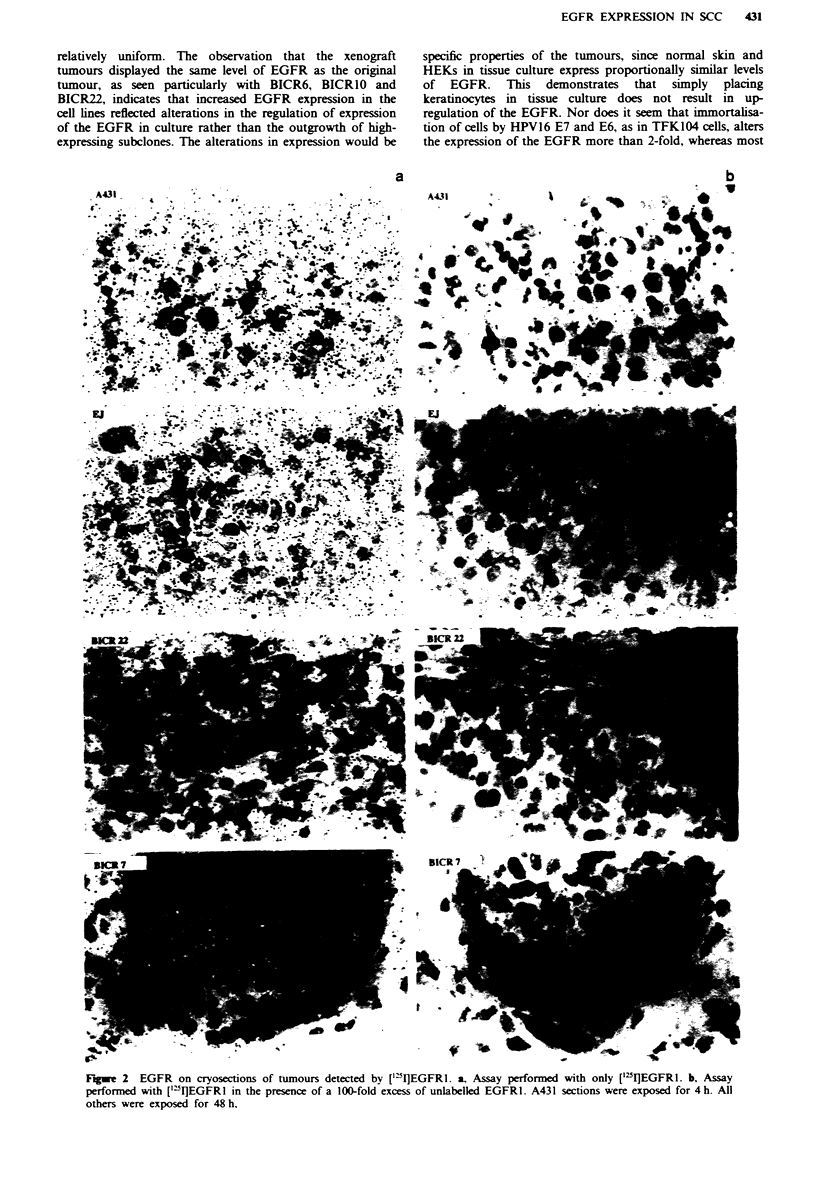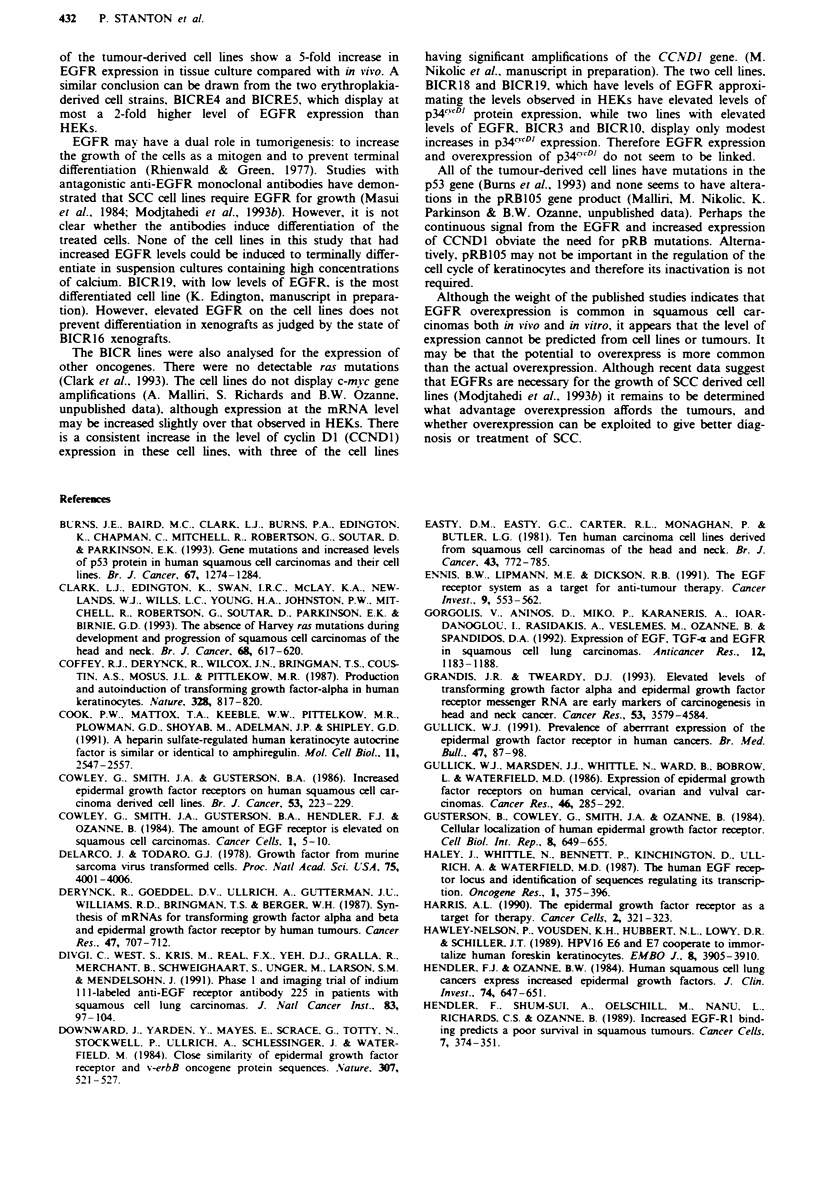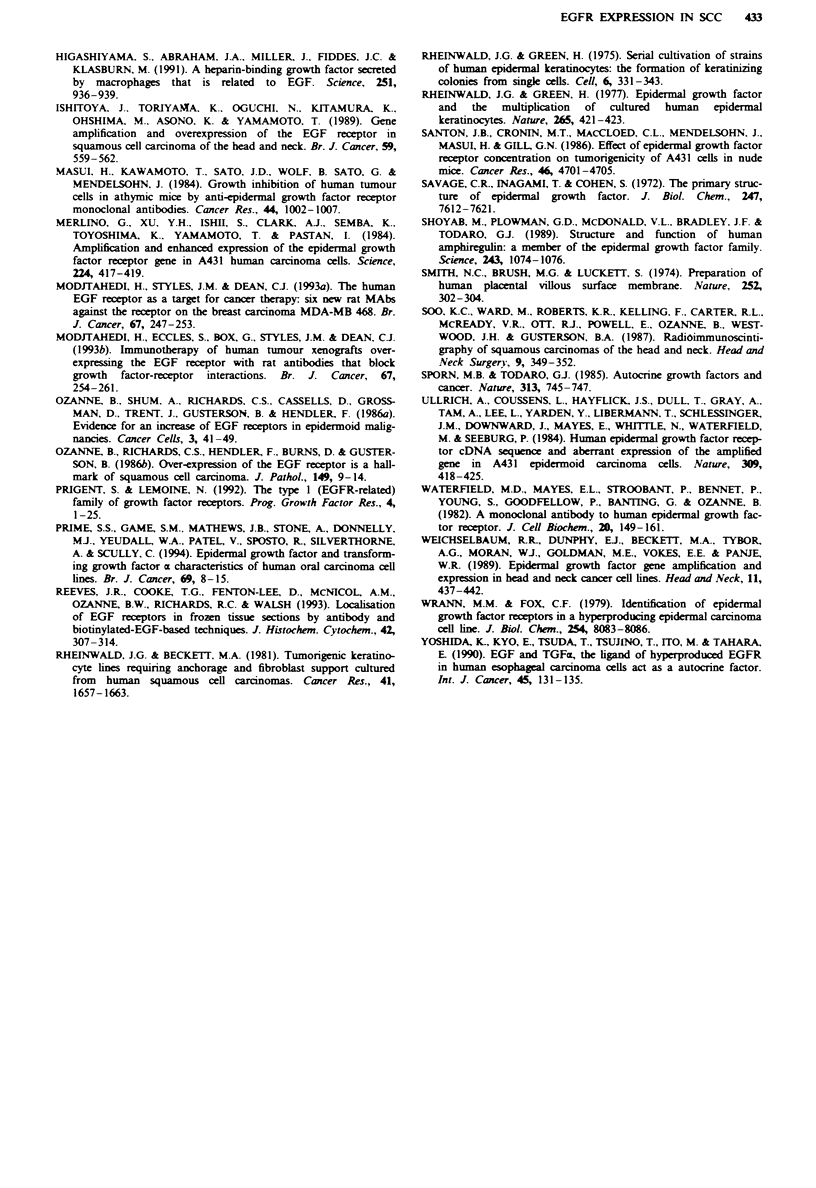# Epidermal growth factor receptor expression by human squamous cell carcinomas of the head and neck, cell lines and xenografts.

**DOI:** 10.1038/bjc.1994.322

**Published:** 1994-09

**Authors:** P. Stanton, S. Richards, J. Reeves, M. Nikolic, K. Edington, L. Clark, G. Robertson, D. Souter, R. Mitchell, F. J. Hendler

**Affiliations:** Department of Surgery, University of Glasgow, Glasgow Royal Infirmary, UK.

## Abstract

**Images:**


Br. J. Cancer (1994), 70, 427-433 © Macmillan Press Ltd., 1994

### Epidermal growth factor receptor expression by human squamous cell carcinomas of the head and neck, cell lines and xenografts

P. Stanton[1], S. Richards[2], J. Reeves[1], M. Nikolic[2], K. Edington[2], L. Clark[2], G. Robertson[3], D. Souter[3], R. Mitchell[4], F.J. Hendler[5], T. Cooke[1], E.K. Parkinson[2] & B.W. Ozanne[2]

[1]*Department of Surgery, University of Glasgow, Glasgow Royal Infirmary, Glasgow, UK;* [2]*CRC Beatson Laboratories Beatson Institute for Cancer Research, Switchback Road, Glasgow, UK;* [3]*Canniesburn Hospital, Switchback Road, Glasgow, UK;* [4]*Department of Oral and Maxiillofacial Surgery, Greenbank Road, Edinburgh, UK;* [5]*The J. Graham Brown Cancer Center, Department of Medicine and Biochemistry, University of Louisville School of Medicine, Louisville VA, Medical Center, Louisville, Kentucky, USA.*

**Summary** Epidermal growth factor receptor (EGFR) overexpression has been associated frequently with squamous cell carcinomas (SCC) and SCC cell lines. In most cases the level of EGFR on the tumours from which the cell lines were derived has not been determined, nor have EGFR levels been determined for xenograft tumours from the cell lines. In this study we determined EGFR expression on a new series of head and neck SCC (SCCHN)-derived cell lines, which were obtained from tumours representing a spectrum of malignant progression, and two cell strains derived from erythroplakia premalignant lesions. The level of EGFR on cell lines was determined by [$^{125}$I]EGF competitive binding assays. EGFR levels on some of the original tumours and xenografts of the cell lines were determined on cryosections by a competitive binding assay based on [$^{125}$I]EGFR1, an EGFR-specific monoclonal antibody. EGFR expression on the tumour cryosections was compared with expression on cryosections of skin and buccal mucosa. Eight of the ten tumour cell lines had elevated EGFR. Two of the tumour-derived cell lines and the two erythroplakia-derived cell strains expressed EGFR at levels similar to that detected on normal keratinocytes in tissue culture. Only two of the tumours overexpressed EGFR when compared with normal tissue. The other tumours had levels similar to that detected on the basal layers of skin or buccal mucosa. The xenografts expressed EGFR, as did the original tumours, even though they were derived from cell lines that displayed significant overexpression of EGFR. This study suggests that most tumours have a latent potential to overexpress EGFR which is realised in tissue culture.

The EGFR is frequently, but not necessarily, overexpressed on SCC-derived cell lines (Wrann & Fox, 1979; Merlino *et al.*, Cowley *et al.*, 1984, 1986; 1984; Ozanne *et al.*, 1986*a*; Prime *et al.*, 1994) and tumours (Hendler & Ozanne, 1984; Gullick *et al.*, 1986; Ozanne *et al.*, 1986*b*; Derynck *et al.*, 1987; Hendler *et al.*, 1989; Ishitoya *et al.*, 1989; Weichselbaum *et al.*, 1989; Gullick, 1991; Gorgolis *et al.*, 1992). Its gene, c-*erb*B (Downward *et al.*, 1984), is located on chromosome 7p12-14 and spans at least 110 kb (Haley *et al.*, 1987). It encodes two mRNAs of 10 and 5 kb (Ullrich *et al.*, 1984). The EGFR is a phosphoglycoprotein of 170 kDa (Prigent & Lemoine, 1992). The extracellular domain binds ligand, which activates the intracellular domain protein tyrosine kinase. The ligands for the EGFR include epidermal growth factor (EGF) (Savage *et al.*, 1972), transforming growth factor alpha (TGF-α) (DeLarco & Todaro, 1978), heparin-binding epidermal growth factor (HBEGF) (Higashiyama *et al.*, 1991) and amphiregulin (AR) (Shoyab *et al.*, 1989). Both non-malignant keratinocytes and squamous cell carcinomas can produce EGF and TGF-α (Coffey *et al.*, 1987; Derynck *et al.*, 1987; Yoshida *et al.*, 1990) and HBEGF (Cook *et al.*, 1991) and thereby may constitute an autocrine system (Sporn & Todaro, 1985). The first indication that overexpression might be important to tumour growth was provided by experiments with the vulval epidermoid carcinoma-derived cell line, A431, which has $2 \times 10^6$ EGFRs per cell. Variants were selected which had fewer EGFRs. These variants were less tumorigenic than the parental A431 cells (Santon *et al.*, 1986). Overexpression of EGFR by SCC cell lines might help them respond to low amounts of EGF or TGF-α, while inhibition of ligand binding to the EGFR by EGFR-specific monoclonal antibodies seems to block the growth of SCC cell lines in tissue culture and xenografts, which highlights the importance of the EGFR to the growth of SCC (Masui *et al.*, 1984; Modjtahedi *et al.*, 1993*a*, *b*). Owing to its reported overexpression, the EGFR has been suggested to be a potential target for diagnosis (Soo *et al.*, 1987; Divgi *et al.*, 1991) or directed therapy of SCC (Harris, 1990; Ennis *et al.*, 1991). Although several studies have indicated that the EGFR is overexpressed on human SCC, quantitation of the overexpression relies heavily on the study of SCC-derived cell lines in which the frequency of significant overexpression is very high (Cowley *et al.*, 1984, 1986). Most studies have relied upon cell lines which have been isolated under culture conditions unfavourable for the growth of normal keratinocytes (Easty *et al.*, 1981). Thus, they may have selected for tumours which express very high levels of EGFR *in vivo*; otherwise only a minority of the tumour cells that overexpress the EGFR would be selected for growth in culture. A recent study which established cell lines from oral cavity tumours found that only one out of eight cell lines overexpressed the EGFR (Prime *et al.*, 1994). In the present study we have analysed EGFR expression on a series of cell lines derived from SCCHN under culture conditions favourable for the growth of normal keratinocytes (Rheinwald & Beckett, 1981). We have also determined EGFR levels on cryosections of the original tumours and on xenografts of some of the cell lines.

### Materials and methods

### *Cell culture*

All cells were cultured at 37°C in a humidified atmosphere of 5% carbon dioxide in air. A431, EJ and ZR-75-1 cells were maintained in Dulbecco's modified Eagle medium (DMEM, Gibco, Paisley, UK), with 10% fetal bovine serum (FBS, Gibco, Paisley, UK), and supplemented with L-glutamine, penicillin and streptomycin.

The HPV16 E7, E6 immortalised human keratinocyte line TFK 104 (Hawely-Nelson *et al.*, 1989) was kindly supplied

Correspondence: B. Ozanne, Beatson Institute for Cancer Research, Garscube Estate, Switchback Road, Bearsden, Glasgow G61 1BD, Scotland, UK.
Received 28 January 1994; and in revised form 25 April 1994.

by K. Vousden (Ludwig Institute for Cancer Research, St Mary's Hospital Medical School, London, UK). Cells were maintained in keratinocyte SFM (Gibco), supplemented with bovine pituitary extract (Clonetics), EGF (10 ng $ml^{-1}$) and antibiotics. Human epidermal keratinocytes (HEKs) were maintained in complete KGM (Clonetics).

BICR squamous cell carcinoma lines listed in Table I (K. Edington *et al.*, manuscript in preparation) were grown on lethally irradiated NIH3T3 feeder cells (Rheinwald & Green, 1975) in DMEM with 10% FBS supplemented with hydrocortisone (0.4 $\mu$g $ml^{-1}$), glutamine and antibiotics. BICR 18 and BICR 22 grow optimally in 2% FBS.

### *EGF receptor binding assay*

[$^{125}$I]EGF (Amersham, Cat. No. IM124, 50 $\mu$Ci $ml^{-1}$) was diluted to a specific activity of 0.05 $\mu$Ci $ng^{-1}$ with unlabelled EGF (mouse, tissue culture grade, Sigma). The assay was performed as described previously (Cowley *et al.*, 1986). Briefly, cells were plated at a density of $1 \times 10^5$ cells per well in 24-well tissue culture plates 24 h before the assay. The wells were washed three times with phosphate-buffered saline (PBS), and incubated with labelled EGF in the presence or absence of a 100-fold excess of unlabelled competitor in 0.5 ml of PBS with 1% BSA at room temperature for 1 h. The range of competitor was 11 points between 1.0 ng and 1 $\mu$g. Cells were then washed five times with ice-cold PBS/5% BSA, solubilised in 1 ml of 1 N sodium hydroxide and the amount of radioactivity determined in a gamma counter. Counts were corrected for background and cell number and data were analysed using the LIGAND program.

### *Binding of EGFR1 and EGF to placental membrane preparations*

EGF and EGFR1 were radioiodinated using iodogen to specific activities of 40 and 250 $\mu$Ci $\mu g^{-1}$ respectively. Human syncytiotrophoblast plasma membranes were prepared by cold isotonic saline extraction (Smith *et al.*, 1974). The assays were performed in 96-well plates at 4°C in total volumes of 300 $\mu$l and the reagents were diluted in PBS/BSA (0.2%) for the EGF assay and with 5% normal human serum for the antibody assay. For the EGF assay 25,000 c.p.m. of [$^{125}$I]EGF with a range of quantities of unlabelled EGF (18 points from 10 pg to 1 $\mu$g) and for EGFR1, 50,000 c.p.m. of [$^{125}$I]EGFR1 with a range of quantities of unlabelled EGFR1 (11 points from 2 ng to 2 $\mu$g) were incubated with 10 $\mu$g of protein of placental membranes for 4 h at 0°C. The membranes were harvested and washed onto glass-fibre filters in a multiple manifold vacuum harvester (LKB).

The radioactivity bound to the filters was determined with a gamma counter. The data from duplicate experiments were pooled and analysed with the LIGAND program to determine receptor concentrations. The EGF binding data indicated two classes of receptors with a combined apparent concentration of 2.3 pmol per mg of placental membrane protein ± 12%. The EGFR1 binding gave only one class of receptor with a concentration of 1.7 pmol per mg of placental membrane protein ± 9%.

### *EGF and EGFR1 immunohistochemistry on skin cryosections*

EGF was biotinylated and detected on skin cryosections (Reeves *et al.*, 1993). EGFR1 was detected using biotinylated rabbit anti-mouse antibody, and non-immune mouse IgG2b was used as a control for non-specific antibody binding. (Reeves *et al.*, 1993).

### *Quantitation of EGFR on cryosections of tumours*

Cryosections were made from frozen tumour or xenograft specimens (K. Edington, manuscript in preparation). The sections were fixed in 50% acetone-PBS. Two sections from each specimen were incubated with 200,000 c.p.m. of [$^{125}$I]EGFR1 (specific activity of $2.5 \times 10^5$ c.p.m. $mg^{-1}$ EGFR1) for 3 h at 24°C in a volume of 100 $\mu$l of 50% calf serum-PBS. Another section was treated similarly but a 200-fold excess of unlabelled EGFR1 was included in the 100 $\mu$l. The sections were washed four times with 10% calf serum-PBS, rinsed with water, air dried and placed in an X-ray cassette and exposed to film for 16 h. The sections were dipped in photographic emulsion (NTB-2 Kodak) and exposed for sufficient time to generate between 100 and 400 grains per 100 $\mu m^2$. The time of exposure was estimated from the density of the autoradiograph. Based on densitometric scanning of the A431 cell pellet sections, the emulsions of A431 require 4 h exposure to give an acceptable grain density. EJ cell pellets or normal skin require 48 h. The silver grains are counted per high-power field (HPF) which equals $6.67 \times 10^{-3}$ $mm^2$ with ten fields per slide using a Joyce-Loebel Nikon Magiscan computer. Using this protocol, approximately equal numbers of grains were counted per sample and expressed as the number of grains 100 $\mu m^{-2}$ $h^{-1}$ exposure after subtracting the background binding.

### Results

### *EGFR levels on the SCCHN cell lines*

Ligand-binding studies have been used to determine the number of EGFRs on many different cell types. Previously it has been determined that normal keratinocytes grown in tissue culture express $2.7 \times 10^5$ EGFRs per cell (Cowley *et al.*, 1984, 1986) or $5.5 \times 10^5$ EGFRs per cell if isolated from gingival mucosa (Prime *et al.*, 1994), while most SCC-derived cell lines express around $10^6$ EGFRs per cell. (Cowley *et al.*, 1984, 1986). We performed [$^{125}$I]EGF binding studies to determine the number of EGFRs per cell on ten SCCHN, two erythroplakia-derived cell lines, HPV16 E7, E6 immortalised foreskin keratinocytes (TFK104) and HEKs (Table I). Eight of the cell lines express from $9 \times 10^5$ to $1.8 \times 10^6$ EGFRs per cell, as do most other SCC-derived cell lines. One of the tumour-derived cell lines, BICR19, and one of the erythroplakia-derived cell lines, BICRE4, showed no increase in EGFR expression compared with HEK, while BICR18 and TFK104 showed a 2-fold increase in EGFRs compared with HEKs. In BICR18, BICR19, BICRE4 and BICRE5 the levels of EGFR were similar to those found in a recent study (Prime *et al.*, 1994). Thus, it is not esssential for all SCCHN-derived cell lines to overexpress EGFR, nor is HPV16 E7 and E6 expression sufficient to cause more than a 2-fold increase in EGFR expression.

**Table I** EGFR expression on cell lines

| *Cell line* | *Origin* | *EGFR × $10^6$ per cell* |
|---|---|---|
| HEK | Epidermal keratinocyte | 0.2 |
| EJ | Transitional cell carcinoma | 0.2 |
| TFK104 | Cervical keratinocyte | 0.4 |
| A431 | Carcinoma, vulva | 1.8 |
| BICRE4 | Erythroplakia | 0.2 |
| BICRE5 | Erythroplakia | 0.3 |
| BICR3 | Alveolus | 1.4 |
| BICR6 | Hypopharynx | 1.8 |
| BICR7 | Tongue | 1.2 |
| BICR10 | Buccal mucosa | 0.9 |
| BICR16 | Tongue | 1.6 |
| BICR18 | Larynx | 0.4 |
| BICR19 | Epidermis | 0.2 |
| BICR22 | Tongue | 1.0 |
| BICR31 | Tongue | 1.2 |
| BICR56 | Tongue | 1.3 |
| BICR63 | Tongue | 0.9 |
| BICR68 | Tongue | 1.0 |

The number of EGFRs per cell was determined from ligand-binding experiments as described in the Materials and methods section. The BICR cell lines were derived by K. Edington and K. Parkinson (unpublished data).

**Figure 1** Localisation of the EGFR on cryosections of human skin. **a**, Biotinylated EGF. **b**, Biotinylated EGF plus 100 × EGF. **c**, EGFR1. **d**, Control MAb. **e**, [125I]EGFR1. **f**, [125I]EGFR1 plus 100 × unlabelled EGFR1.

*Determination of EGFR expression on tumour biopsy specimens*

To determine if EGFR overexpression on the cell lines reflected the level of EGFR on the tumours from which they were derived, we measured EGFR expression on tumour cryosections. To quantitate the level of EGFR we used an assay based on the competitive binding of the EGFR-specific monoclonal antibody, EGFR1 (Waterfield *et al.*, 1982), to cryosections of tumour biopsy specimens (Gusterson *et al.*, 1984; Hendler & Ozanne, 1984; Ozanne *et al.*, 1986; Hendler *et al.*, 1989). To demonstrate the specificity of EGFR1, we observed the binding of EGF (Figure 1a and b) and EGFR1 (Figure 1c and d) to cryosections of normal skin. This demonstrates that EGF and EGFR1 binding results in the same pattern of staining. Immunohistochemical staining is not quantitative. As the quantitation technique measures binding of [125I]EGFR1 by the counting of silver grains following autoradiography we wished to demonstrate that [125I]EGFR1 resulted in the same pattern of binding to skin cryosections as did unlabelled EGFR1. Cryosections of skin were exposed to [125I]EGFR1 in the presence or absence of an excess of unlabelled EGFR1 (Figure 1e and f). The distribution of the grains generated by radiolabelled EGFR1 was reminiscent of the pattern of staining observed using biotinylated EGF or EGFR1 detected immunohistochemically. Competition with a 100-fold excess of unlabelled EGFR1 demonstrated that binding of [125I]EGFR1 was specific. These observations provided the basis for the quantitation of EGFR on biopsy sections.

To demonstrate that the competitive binding assay was quantitative we used several assays. Firstly, we determined the concentration of EGFR in placental membrane preparations using either [125I]EGF or [125I]EGFR1 (Table II). EGFR determined with both ligands was very similar. Secondly, we compared the EGFR levels in cell lines with known numbers of EGFR, A431, EJ and ZR-75-1 (Hendler & Ozanne, 1984; Ozanne *et al.*, 1986), by counting the silver grains on cryosections of cell pellets of the cell lines (Table III). Scatchard analysis indicated that A431 cells displayed approximately eight times more EGFR than did EJ cells (Ozanne *et al.*, 1986; Hendler *et al.*, 1989), while ZR-75-1 cells have only $5 \times 10^3$ EGFRs per cell. EGFR1 binding as determined by silver grain counts yielded similar results in terms of the difference in binding between A431, EJ and ZR-75-1 cell lines. Thirdly, we compared specific binding of EGFR1 and EGF to cryosections of skin and EJ cell pellets. Ligand-binding assays indicated that both HEKs and EJ cells have around $2.5 \times 10^5$ EGFRs per cell. The EGFR1-EGF ratio of grains on EJ cell pellets and skin was 1.5 and 1.6 respectively (Table IV). For an assay to be comparable the binding of EGFR1 to A431 and EJ cell pellets has to differ by 6- to 8-fold and the binding to frozen sections of skin must be within the range of binding to EJ cells.

**Table II** Quantitation of EGFR in placental membranes

| Ligand | pM EGFR $mg^{-1}$ protein |
|---|---|
| EGF | 2.3 ± 12% |
| EGFR1 | 1.7 ± 9% |

Binding assays were performed on placental membrane preparations. The data represent the average of two independent analyses as described in the Materials and methods section.

**Table III** Binding of EGF and EGFR1 to cell lines

| Cell line | EGFR per cell | Ratio to EJ | EGFR1 | Ratio to EJ |
|---|---|---|---|---|
| A431 | $1.6 \times 10^6$ | 8 | 26431 | 7.15 |
| EJ | $2.0 \times 10^5$ | 1 | 3698 | 1.0 |
| ZR75 | $5 \times 10^3$ | 0.004 | 97 | 0.0026 |

[$^{125}$I]EGFR1 was used to determine the relative binding to cell lines which differed in the level of expression of EGFR. Numbers represent specific binding of [$^{125}$I]EGFR1 to cryosections of frozen pellets of the cells. The numbers represent the net silver grains per ten high-power fields per hour of each section. Two cryosections of each cell line were counted.

**Table IV** EGF and EGFR1 binding to cryosections of skin and EJ cells

| Section | EGFR1 | Area | C/A | Hours | C/A $h^{-1}$ |
|---|---|---|---|---|---|
| EGFR1 | | | | | |
| EJ-1 | 2705 | 6.67 | 406 | 50 | 8.1 |
| EJ-2 | 3254 | 6.67 | 488 | 50 | 9.8 |
| Skin 1 | 1867 | 1.56 | 1197 | 194 | 6.2 |
| Skin 2 | 887 | 0.75 | 1183 | 194 | 6.1 |
| EGF | | | | | |
| EJ-1 | 3286 | 6.67 | 493 | 96 | 5.1 |
| Skin 1 | 1768 | 2.81 | 629 | 194 | 3.2 |

Ratio EJ/skin: EGFR1, 1.5; EGF, 1.6. The sections were treated as described in the Materials and methods section. The numbers represent grain counts per 100 $\mu m^2$. The non-specific binding was subtracted. The exposure times were determined from 16 h exposures of autoradiographs of the slides.

**Table V** Binding of EGFR1 to tumour and xenograft cryosections

| Tumour | Net counts | Ratio of tumour to skin | Ratio of xenograft to skin |
|---|---|---|---|
| A431 | 22560 | 7.34 | NA |
| Skin | 3073 | 1.00 | NA |
| BM | 3780 | 1.23 | NA |
| EJ | 2854 | 0.93 | NA |
| BICR3 | 3685 | 1.20 | ND |
| BICR6 | 10037 | 3.27 | 3.46 |
| BICR7 | 994 | 0.32 | ND |
| BICR10 | 4794 | 1.56 | 1.37 |
| BICR16 | 3763 | 1.22 | 0.02 |
| BICR18 | 5674 | 1.85 | ND |
| BICR19 | 2714 | 0.88 | 0.07 |
| BICR22 | 1090 | 0.35 | 0.89 |

Measurement of [$^{125}$]EGFR1 binding to cryosections of cell pellets for the cell lines and biopsy specimens for skin, buccal mucosa (BM), tumours and xenografts. All slides were exposed for 24 h, except A431, which were normalised to 24 h from 4.5 h. The counts represent the net silver grains per ten high-power fields.

Analysis of cryosections of the tumours (where available) from which the BICR cell lines listed in Table I were derived was performed using [$^{125}$I]EGFR1. Silver grains are localised above normal skin (Figure 1d), A431 (Figure 2) and EJ (Figure 2) cell pellets and two of the tumours, BICR7 (Figure 2) and BICR22 (Figure 2). Quantitation of the grains indicates that only two of the tumours displayed elevated levels of EGFR, BICR6 and BICR18 (Table V). The increase in EGFR expression in these tumours was higher than in normal skin, but the difference was not as great as that between the cell lines and HEKs (Table I). The other tumours from which cell lines were derived which have significant increases in EGFR expression did not have a significant increase in EGFR compared with normal skin. In fact one of them, BICR22, displayed a decrease in the actual tumour, while the cell line had a 5-fold increase. The cell lines which displayed slightly elevated or HEK levels of EGFR, BICR18 and BICR19 respectively, were derived from tumours with slightly elevated or skin levels of EGFR respectively. This indicates that the EGFR1-binding assay does not give artificially low levels of EGFR on the tumour sections.

The data above suggested that the increased expression of EGFR observed on the BICR lines is a property of a subset of tumour cells that grow in tissue culture and does not reflect the state of expression in the original tumour. An alternative explanation is that the potential to overexpress the EGFR is suppressed in the tumours. To evaluate the alternative explanation, we analysed xenograft tumours of some of the cell lines isolated from nude mice. BICR6, BICR10 and BICR22 expressed essentially the same level of EGFR as they did in the tumour biopsy assays (Table V). Binding of EGFR1 to the xenografts for BICR16 and BICR19 was almost non-existent, which may reflect the highly differentiated state of these xenografts (data not shown).

### Discussion

Using EGF ligand-binding assays we have demonstrated that 80% of a series of cell lines derived from SCCHN had a significant increase in the level of expression of EGFR, consistent with most previously reported studies (Cowley *et al.*, 1984, 1986; Ozanne *et al.*, 1986; Hendler *et al.*, 1989; Weichselbaum *et al.*, 1989). In two of the cell lines, however, EGFR expression was similar to that normally seen in HEKs maintained in tissue culture. Two cell strains derived from erythroplakias did not have elevated levels of EGFR. This suggests that increased EGFR expression is not necessarily associated with premalignant lesions as both lesions progressed to SCC within a year (K. Edington *et al.*, manuscript in preparation). Furthermore, both BICRE4 and BICRE5 are defective for terminal differentiation when placed in suspension culture, which is consistent with their representing premalignant keratinocytes. Recently it has been suggested that expression of the EGFR and TGF-α expression is increased in dysplastic lesions adjacent to SCCHN (Grandis & Tweardy, 1993). The fact that the DOK cell line, derived from such a lesion, displays a 3-fold increase in EGFR expression (data not shown) as measured by ligand binding is consistent with this observation. The BICRE4 and BICRE5 cell lines may represent a distinct pathway occurring before malignancy has developed. Although the majority of cell lines were derived from late-stage tumours, metastases and recurrences, two cell lines, BICR3 and BICR63, were derived from T2 stage tumours and were found to have elevated levels of EGFR, suggesting that increased levels of expression are not limited to cell lines derived from late-stage tumours. However, as one of the cell lines, BICR18, derived from a recurrent tumour, and another, BICR19, derived from a large and aggressive skin tumour, had normal levels of EGFR expression, overexpression as detected in cultured cells is not necessary for SCC progression.

The cell lines in this analysis were established and maintained using 3T3 feeder layers under culture conditions as close as possible to those that would support the growth of normal keratinocytes yet give optimal growth of the tumour cells (Rheinwald & Beckett, 1981; K. Edington, manuscript in preparation). This was done in an attempt to establish cell lines representing the cells in the original tumour as closely as possible. It is possible that even under these culture conditions cells capable of overexpressing the EGFR were selected. From the analysis of the EGFR1 binding to the cryosections of the original tumours, no focal expression of the EGFR, which would have suggested a subpopulation of cells with high level of EGFR expression within the tumour, was observed. Rather, the distribution of antibody seemed to be relatively uniform. The observation that the xenograft tumours displayed the same level of EGFR as the original tumour, as seen particularly with BICR6, BICR10 and BICR22, indicates that increased EGFR expression in the cell lines reflected alterations in the regulation of expression of the EGFR in culture rather than the outgrowth of high-expressing subclones. The alterations in expression would be specific properties of the tumours, since normal skin and HEKs in tissue culture express proportionally similar levels of EGFR. This demonstrates that simply placing keratinocytes in tissue culture does not result in up-regulation of the EGFR. Nor does it seem that immortalisation of cells by HPV16 E7 and E6, as in TFK104 cells, alters the expression of the EGFR more than 2-fold, whereas most

**Figure 2** EGFR on cryosections of tumours detected by [$^{125}$I]EGFR1. **a**, Assay performed with only [$^{125}$I]EGFR1. **b**, Assay performed with [$^{125}$I]EGFR1 in the presence of a 100-fold excess of unlabelled EGFR1. A431 sections were exposed for 4 h. All others were exposed for 48 h.

of the tumour-derived cell lines show a 5-fold increase in EGFR expression in tissue culture compared with *in vivo*. A similar conclusion can be drawn from the two erythroplakia-derived cell strains, BICRE4 and BICRE5, which display at most a 2-fold higher level of EGFR expression than HEKs.

EGFR may have a dual role in tumorigenesis: to increase the growth of the cells as a mitogen and to prevent terminal differentiation (Rhienwald & Green, 1977). Studies with antagonistic anti-EGFR monoclonal antibodies have demonstrated that SCC cell lines require EGFR for growth (Masui *et al.*, 1984; Modjtahedi *et al.*, 1993*b*). However, it is not clear whether the antibodies induce differentiation of the treated cells. None of the cell lines in this study that had increased EGFR levels could be induced to terminally differentiate in suspension cultures containing high concentrations of calcium. BICR19, with low levels of EGFR, is the most differentiated cell line (K. Edington, manuscript in preparation). However, elevated EGFR on the cell lines does not prevent differentiation in xenografts as judged by the state of BICR16 xenografts.

The BICR lines were also analysed for the expression of other oncogenes. There were no detectable *ras* mutations (Clark *et al.*, 1993). The cell lines do not display c-*myc* gene amplifications (A. Malliri, S. Richards and B.W. Ozanne, unpublished data), although expression at the mRNA level may be increased slightly over that observed in HEKs. There is a consistent increase in the level of cyclin D1 (CCND1) expression in these cell lines, with three of the cell lines having significant amplifications of the *CCND1* gene. (M. Nikolic *et al.*, manuscript in preparation). The two cell lines, BICR18 and BICR19, which have levels of EGFR approximating the levels observed in HEKs have elevated levels of $p34^{cycD1}$ protein expression, while two lines with elevated levels of EGFR, BICR3 and BICR10, display only modest increases in $p34^{cycD1}$ expression. Therefore EGFR expression and overexpression of $p34^{cycD1}$ do not seem to be linked.

All of the tumour-derived cell lines have mutations in the p53 gene (Burns *et al.*, 1993) and none seems to have alterations in the pRB105 gene product (Malliri, M. Nikolic, K. Parkinson & B.W. Ozanne, unpublished data). Perhaps the continuous signal from the EGFR and increased expression of CCND1 obviate the need for pRB mutations. Alternatively, pRB105 may not be important in the regulation of the cell cycle of keratinocytes and therefore its inactivation is not required.

Although the weight of the published studies indicates that EGFR overexpression is common in squamous cell carcinomas both *in vivo* and *in vitro*, it appears that the level of expression cannot be predicted from cell lines or tumours. It may be that the potential to overexpress is more common than the actual overexpression. Although recent data suggest that EGFRs are necessary for the growth of SCC derived cell lines (Modjtahedi *et al.*, 1993*b*) it remains to be determined what advantage overexpression affords the tumours, and whether overexpression can be exploited to give better diagnosis or treatment of SCC.

### References

BURNS, J.E., BAIRD, M.C., CLARK, L.J., BURNS, P.A., EDINGTON, K., CHAPMAN, C., MITCHELL, R., ROBERTSON, G., SOUTAR, D. & PARKINSON, E.K. (1993). Gene mutations and increased levels of p53 protein in human squamous cell carcinomas and their cell lines. *Br. J. Cancer*, **67**, 1274-1284.

CLARK, L.J., EDINGTON, K., SWAN, I.R.C., MCLAY, K.A., NEWLANDS, W.J., WILLS, L.C., YOUNG, H.A., JOHNSTON, P.W., MITCHELL, R., ROBERTSON, G., SOUTAR, D., PARKINSON, E.K. & BIRNIE, G.D. (1993). The absence of Harvey *ras* mutations during development and progression of squamous cell carcinomas of the head and neck. *Br. J. Cancer*, **68**, 617-620.

COFFEY, R.J., DERYNCK, R., WILCOX, J.N., BRINGMAN, T.S., COUSTIN, A.S., MOSUS, J.L. & PITTLEKOW, M.R. (1987). Production and autoinduction of transforming growth factor-alpha in human keratinocytes. *Nature*, **328**, 817-820.

COOK, P.W., MATTOX, T.A., KEEBLE, W.W., PITTELKOW, M.R., PLOWMAN, G.D., SHOYAB, M., ADELMAN, J.P. & SHIPLEY, G.D. (1991). A heparin sulfate-regulated human keratinocyte autocrine factor is similar or identical to amphiregulin. *Mol. Cell Biol.*, **11**, 2547-2557.

COWLEY, G., SMITH, J.A. & GUSTERSON, B.A. (1986). Increased epidermal growth factor receptors on human squamous cell carcinoma derived cell lines. *Br. J. Cancer*, **53**, 223-229.

COWLEY, G., SMITH, J.A., GUSTERSON, B.A., HENDLER, F.J. & OZANNE, B. (1984). The amount of EGF receptor is elevated on squamous cell carcinomas. *Cancer Cells*, **1**, 5-10.

DELARCO, J. & TODARO, G.J. (1978). Growth factor from murine sarcoma virus transformed cells. *Proc. Natl Acad. Sci. USA*, **75**, 4001-4006.

DERYNCK, R., GOEDDEL, D.V., ULLRICH, A., GUTTERMAN, J.U., WILLIAMS, R.D., BRINGMAN, T.S. & BERGER, W.H. (1987). Synthesis of mRNAs for transforming growth factor alpha and beta and epidermal growth factor receptor by human tumours. *Cancer Res.*, **47**, 707-712.

DIVGI, C., WEST, S., KRIS, M., REAL, F.X., YEH, D.J., GRALLA, R., MERCHANT, B., SCHWEIGHAART, S., UNGER, M., LARSON, S.M. & MENDELSOHN, J. (1991). Phase 1 and imaging trial of indium 111-labeled anti-EGF receptor antibody 225 in patients with squamous cell lung carcinomas. *J. Natl Cancer Inst.*, **83**, 97-104.

DOWNWARD, J., YARDEN, Y., MAYES, E., SCRACE, G., TOTTY, N., STOCKWELL, P., ULLRICH, A., SCHLESSINGER, J. & WATERFIELD, M. (1984). Close similarity of epidermal growth factor receptor and v-*erbB* oncogene protein sequences. *Nature*, **307**, 521-527.

EASTY, D.M., EASTY, G.C., CARTER, R.L., MONAGHAN, P. & BUTLER, L.G. (1981). Ten human carcinoma cell lines derived from squamous cell carcinomas of the head and neck. *Br. J. Cancer*, **43**, 772-785.

ENNIS, B.W., LIPMANN, M.E. & DICKSON, R.B. (1991). The EGF receptor system as a target for anti-tumour therapy. *Cancer Invest.*, **9**, 553-562.

GORGOLIS, V., ANINOS, D., MIKO, P., KARANERIS, A., IOARDANOGLOU, I., RASIDAKIS, A., VESLEMES, M., OZANNE, B. & SPANDIDOS, D.A. (1992). Expression of EGF, TGF-α and EGFR in squamous cell lung carcinomas. *Anticancer Res.*, **12**, 1183-1188.

GRANDIS, J.R. & TWEARDY, D.J. (1993). Elevated levels of transforming growth factor alpha and epidermal growth factor receptor messenger RNA are early markers of carcinogenesis in head and neck cancer. *Cancer Res.*, **53**, 3579-4584.

GULLICK, W.J. (1991). Prevalence of aberrant expression of the epidermal growth factor receptor in human cancers. *Br. Med. Bull.*, **47**, 87-98.

GULLICK, W.J., MARSDEN, J.J., WHITTLE, N., WARD, B., BOBROW, L. & WATERFIELD, M.D. (1986). Expression of epidermal growth factor receptors on human cervical, ovarian and vulval carcinomas. *Cancer Res.*, **46**, 285-292.

GUSTERSON, B., COWLEY, G., SMITH, J.A. & OZANNE, B. (1984). Cellular localization of human epidermal growth factor receptor. *Cell Biol. Int. Rep.*, **8**, 649-655.

HALEY, J., WHITTLE, N., BENNETT, P., KINCHINGTON, D., ULLRICH, A. & WATERFIELD, M.D. (1987). The human EGF receptor locus and identification of sequences regulating its transcription. *Oncogene Res.*, **1**, 375-396.

HARRIS, A.L. (1990). The epidermal growth factor receptor as a target for therapy. *Cancer Cells*, **2**, 321-323.

HAWLEY-NELSON, P., VOUSDEN, K.H., HUBBERT, N.L., LOWY, D.R. & SCHILLER, J.T. (1989). HPV16 E6 and E7 cooperate to immortalize human foreskin keratinocytes. *EMBO J.*, **8**, 3905-3910.

HENDLER, F.J. & OZANNE, B.W. (1984). Human squamous cell lung cancers express increased epidermal growth factors. *J. Clin. Invest.*, **74**, 647-651.

HENDLER, F., SHUM-SUI, A., OELSCHILL, M., NANU, L., RICHARDS, C.S. & OZANNE, B. (1989). Increased EGF-R1 binding predicts a poor survival in squamous tumours. *Cancer Cells*, **7**, 374-351.

HIGASHIYAMA, S., ABRAHAM, J.A., MILLER, J., FIDDES, J.C. & KLASBURN, M. (1991). A heparin-binding growth factor secreted by macrophages that is related to EGF. *Science*, **251**, 936-939.

ISHITOYA, J., TORIYAMA, K., OGUCHI, N., KITAMURA, K., OHSHIMA, M., ASONO, K. & YAMAMOTO, T. (1989). Gene amplification and overexpression of the EGF receptor in squamous cell carcinoma of the head and neck. *Br. J. Cancer*, **59**, 559-562.

MASUI, H., KAWAMOTO, T., SATO, J.D., WOLF, B. SATO, G. & MENDELSOHN, J. (1984). Growth inhibition of human tumour cells in athymic mice by anti-epidermal growth factor receptor monoclonal antibodies. *Cancer Res.*, **44**, 1002-1007.

MERLINO, G., XU, Y.H., ISHII, S., CLARK, A.J., SEMBA, K., TOYOSHIMA, K., YAMAMOTO, T. & PASTAN, I. (1984). Amplification and enhanced expression of the epidermal growth factor receptor gene in A431 human carcinoma cells. *Science*, **224**, 417-419.

MODJTAHEDI, H., STYLES, J.M. & DEAN, C.J. (1993*a*). The human EGF receptor as a target for cancer therapy: six new rat MAbs against the receptor on the breast carcinoma MDA-MB 468. *Br. J. Cancer*, **67**, 247-253.

MODJTAHEDI, H., ECCLES, S., BOX, G., STYLES, J.M. & DEAN, C.J. (1993*b*). Immunotherapy of human tumour xenografts over-expressing the EGF receptor with rat antibodies that block growth factor-receptor interactions. *Br. J. Cancer*, **67**, 254-261.

OZANNE, B., SHUM, A., RICHARDS, C.S., CASSELLS, D., GROSSMAN, D., TRENT, J., GUSTERSON, B. & HENDLER, F. (1986*a*). Evidence for an increase of EGF receptors in epidermoid malignancies. *Cancer Cells*, **3**, 41-49.

OZANNE, B., RICHARDS, C.S., HENDLER, F., BURNS, D. & GUSTERSON, B. (1986*b*). Over-expression of the EGF receptor is a hallmark of squamous cell carcinoma. *J. Pathol.*, **149**, 9-14.

PRIGENT, S. & LEMOINE, N. (1992). The type 1 (EGFR-related) family of growth factor receptors. *Prog. Growth Factor Res.*, **4**, 1-25.

PRIME, S.S., GAME, S.M., MATHEWS, J.B., STONE, A., DONNELLY, M.J., YEUDALL, W.A., PATEL, V., SPOSTO, R., SILVERTHORNE, A. & SCULLY, C. (1994). Epidermal growth factor and transforming growth factor α characteristics of human oral carcinoma cell lines. *Br. J. Cancer*, **69**, 8-15.

REEVES, J.R., COOKE, T.G., FENTON-LEE, D., MCNICOL, A.M., OZANNE, B.W., RICHARDS, R.C. & WALSH (1993). Localisation of EGF receptors in frozen tissue sections by antibody and biotinylated-EGF-based techniques. *J. Histochem. Cytochem.*, **42**, 307-314.

RHEINWALD, J.G. & BECKETT, M.A. (1981). Tumorigenic keratinocyte lines requiring anchorage and fibroblast support cultured from human squamous cell carcinomas. *Cancer Res.*, **41**, 1657-1663.

RHEINWALD, J.G. & GREEN, H. (1975). Serial cultivation of strains of human epidermal keratinocytes: the formation of keratinizing colonies from single cells. *Cell*, **6**, 331-343.

RHEINWALD, J.G. & GREEN, H. (1977). Epidermal growth factor and the multiplication of cultured human epidermal keratinocytes. *Nature*, **265**, 421-423.

SANTON, J.B., CRONIN, M.T., MACCLOED, C.L., MENDELSOHN, J., MASUI, H. & GILL, G.N. (1986). Effect of epidermal growth factor receptor concentration on tumorigenicity of A431 cells in nude mice. *Cancer Res.*, **46**, 4701-4705.

SAVAGE, C.R., INAGAMI, T. & COHEN, S. (1972). The primary structure of epidermal growth factor. *J. Biol. Chem.*, **247**, 7612-7621.

SHOYAB, M., PLOWMAN, G.D., MCDONALD, V.L., BRADLEY, J.F. & TODARO, G.J. (1989). Structure and function of human amphiregulin: a member of the epidermal growth factor family. *Science*, **243**, 1074-1076.

SMITH, N.C., BRUSH, M.G. & LUCKETT, S. (1974). Preparation of human placental villous surface membrane. *Nature*, **252**, 302-304.

SOO, K.C., WARD, M., ROBERTS, K.R., KELLING, F., CARTER, R.L., MCREADY, V.R., OTT, R.J., POWELL, E., OZANNE, B., WESTWOOD, J.H. & GUSTERSON, B.A. (1987). Radioimmunoscintigraphy of squamous carcinomas of the head and neck. *Head and Neck Surgery*, **9**, 349-352.

SPORN, M.B. & TODARO, G.J. (1985). Autocrine growth factors and cancer. *Nature*, **313**, 745-747.

ULLRICH, A., COUSSENS, L., HAYFLICK, J.S., DULL, T., GRAY, A., TAM, A., LEE, L., YARDEN, Y., LIBERMANN, T., SCHLESSINGER, J.M., DOWNWARD, J., MAYES, E., WHITTLE, N., WATERFIELD, M. & SEEBURG, P. (1984). Human epidermal growth factor receptor cDNA sequence and aberrant expression of the amplified gene in A431 epidermoid carcinoma cells. *Nature*, **309**, 418-425.

WATERFIELD, M.D., MAYES, E.L., STROOBANT, P., BENNET, P., YOUNG, S., GOODFELLOW, P., BANTING, G. & OZANNE, B. (1982). A monoclonal antibody to human epidermal growth factor receptor. *J. Cell Biochem.*, **20**, 149-161.

WEICHSELBAUM, R.R., DUNPHY, E.J., BECKETT, M.A., TYBOR, A.G., MORAN, W.J., GOLDMAN, M.E., VOKES, E.E. & PANJE, W.R. (1989). Epidermal growth factor gene amplification and expression in head and neck cancer cell lines. *Head and Neck*, **11**, 437-442.

WRANN, M.M. & FOX, C.F. (1979). Identification of epidermal growth factor receptors in a hyperproducing epidermal carcinoma cell line. *J. Biol. Chem.*, **254**, 8083-8086.

YOSHIDA, K., KYO, E., TSUDA, T., TSUJINO, T., ITO, M. & TAHARA, E. (1990). EGF and TGFα, the ligand of hyperproduced EGFR in human esophageal carcinoma cells act as a autocrine factor. *Int. J. Cancer*, **45**, 131-135.